# Optimizations of In Vitro Mucus and Cell Culture Models to Better Predict In Vivo Gene Transfer in Pathological Lung Respiratory Airways: Cystic Fibrosis as an Example

**DOI:** 10.3390/pharmaceutics13010047

**Published:** 2020-12-31

**Authors:** Rosy Ghanem, Véronique Laurent, Philippe Roquefort, Tanguy Haute, Sophie Ramel, Tony Le Gall, Thierry Aubry, Tristan Montier

**Affiliations:** 1 Univ Brest, INSERM, EFS, UMR 1078, GGB, F-29200 Brest, France; Rosy.Ghanem@univ-brest.fr (R.G.); Veronique.Laurent@univ-brest.fr (V.L.); Tanguy.Haute@univ-brest.fr (T.H.); Tony.Legall@univ-brest.fr (T.L.G.); 2 IRDL UMR CNRS 6027, Université de Bretagne Occidentale, UFR Sciences et Techniques, 6, Avenue Victor Le Gorgeu CS 93837, CEDEX 3, 29238 Brest, France; Philippe.Roquefort@univ-brest.fr (P.R.); Thierry.Aubry@univ-brest.fr (T.A.); 3 Centre de Ressources et de Compétences de la Mucoviscidose, Fondation Ildys, Presqu’île de Perharidy, 29680 Roscoff, France; Sophie.Ramel@ildys.org; 4 CHRU de Brest, Service de Génétique Médicale et Biologie de la Reproduction, Centre de Référence des Maladies Rares “Maladies Neuromusculaires”, F-29200 Brest, France

**Keywords:** cystic fibrosis, gene delivery, in vitro model, mucus, airway epithelium

## Abstract

The respiratory epithelium can be affected by many diseases that could be treated using aerosol gene therapy. Among these, cystic fibrosis (CF) is a lethal inherited disease characterized by airways complications, which determine the life expectancy and the effectiveness of aerosolized treatments. Beside evaluations performed under in vivo settings, cell culture models mimicking in vivo pathophysiological conditions can provide complementary insights into the potential of gene transfer strategies. Such models must consider multiple parameters, following the rationale that proper gene transfer evaluations depend on whether they are performed under experimental conditions close to pathophysiological settings. In addition, the mucus layer, which covers the epithelial cells, constitutes a physical barrier for gene delivery, especially in diseases such as CF. Artificial mucus models featuring physical and biological properties similar to CF mucus allow determining the ability of gene transfer systems to effectively reach the underlying epithelium. In this review, we describe mucus and cellular models relevant for CF aerosol gene therapy, with a particular emphasis on mucus rheology. We strongly believe that combining multiple pathophysiological features in single complex cell culture models could help bridge the gaps between in vitro and in vivo settings, as well as viral and non-viral gene delivery strategies.

## 1. Introduction

### 1.1. Cystic Fibrosis and Current Protein Treatment

**Figure 1:**
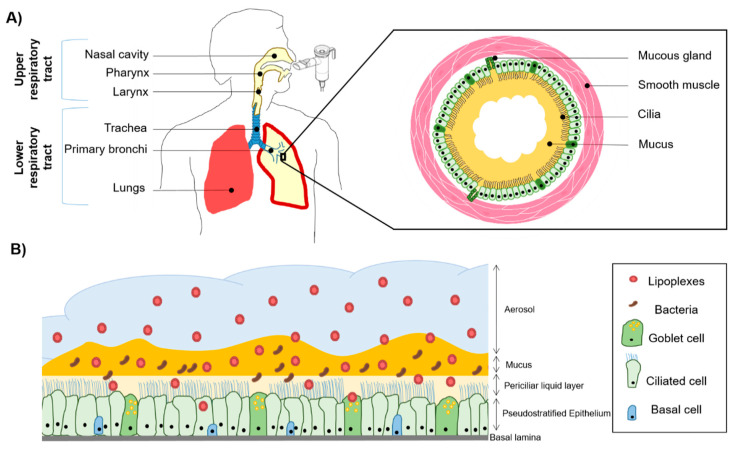
Schematic representation of the respiratory system and the use of an aerosol to target the pulmonary epithelium. (**A**) The upper and lower respiratory tract with an emphasis on a bronchial section constituted by mucous glands and smooth muscles. In the case of cystic fibrosis (CF), sticky mucus is overproduced and creates a bronchial plug. (**B**) Pseudostratified airway epithelium composed of goblet, ciliated, and basal cells. The mucosa is covered with airways surface liquid (ASL) which contains two layers, a periciliary liquid layer directly in contact with the cilia and mucus layer. CF mucus is colonized with opportunistic bacteria such as *Staphylococcus aureus* and *Pseudomonas aeruginosa*. These bacteria form a biofilm participating in the viscous and dense aspect of the CF mucus. When nanoparticles (NPs) such as lipoplexes are aerosolized, they have to cross the mucus barrier before reaching the epithelial cells to deliver therapeutic compounds such as acids nucleic construction.

Cystic fibrosis (CF) is one of the most common inherited genetic disorders, affecting over 70,000 people worldwide [1]. CF is induced by numerous mutations listed in a single gene encoding the cystic fibrosis transmembrane conductance regulator (CFTR) [2]. The CFTR protein is a channel that participates in the exit of chloride ions from the cytoplasm. This channel also plays a key role in the regulation of another channel called ENaC (epithelial sodium channel), involved in the reabsorption of sodium. Altogether, those channels control the correct hydration of mucus on airway epithelial cells. Alteration or dysfunction of the CFTR channel leads thus to abnormal ion transport and mucus dehydration (Figure 1A). Such dehydrated sticky mucus is responsible for the diminution of mucociliary clearance (MCC), leading to a mucosal accumulation. This mucus stagnation provides a propitious environment for bacterial colonization and infections, inducing related chronic inflammation. In the CF context, polynuclear neutrophils accumulation in the mucus is responsible for the production and the excretion of lysosomal enzymes which directly contribute to tissue remodeling [3,4]. The remodeling of secretory cells induces an overproduction of mucin, the main constituent of mucus, leading to severe consequences for the respiratory exchanges [5].

Today, pulmonary complications are the primary cause of CF-related morbidity and mortality. Similar manifestations exist in the gastrointestinal and genital tracts but are, at present, dealt with by symptomatic treatments such as supplementary pancreatic enzymes [6]. During the last decade, new drugs have been developed regarding the CFTR mutations. Ivacaftor (Kalydeco^®^), a CFTR potentiator channel, is indicated for the G551D mutation. This potentiator can be associated with correctors such as lumacaftor (Orkambi^®^) or tezacaftor (Symdeko^®^) for the homozygote F508del mutation. More recently, a tritherapy, namely, elexacaftor-tezacaftor-ivacaftor (Trikafta^®^), showed an FEV1 (forced expiratory volume) higher than 10% benefit for patients with a single F508del allele [7]. Unfortunately, these molecules are mutation-dependent and are sometimes responsible for several side effects (such as hepatitis, abdominal pain) [8]. Even though pharmacology using CFTR potentiators is a real hope for most CF patients, rare mutations cannot currently be treated with those kinds of molecules.

### 1.2. Gene Therapy in CF Disease

Gene therapy may be a relevant approach to treat CF patients whatever their genotype. This technique was primarily used during the first trials of gene therapy in 1989–1990, which were coordinated by the NIH Clinical Center in Bethesda (Maryland) for adenosine deaminase (ADA) deficiency [9]. Since then, this technology kept on developing and became more and more efficient. The principle of gene transfer is based on the introduction of nucleic acids sequences. The plasmid, pDNA, containing a transgene encoding for the gene of interest, can be inserted into targeted cells through different systems. Viral vectors were the first to be used and have already proven efficient. In 2017, gene therapy was proved to be efficient in treating patients with hemophilia A and B using viral AAV vectors, providing a breakthrough to cure these monogenetic diseases [10,11]. The last gene therapy approved by the FDA and the EMA is Zolgensma (Novartis) for the treatment of spinal muscular atrophy [12]. However, viral vectors present some disadvantages. For instance, their immunogenicity limits the possibility of re-administration and they are difficult to produce and to purify. On the other hand, non-viral vectors are based on the use of chemical compounds, mostly lipids or cationic polymers, which directly cross the cytoplasmic membrane of the cells [13,14]. Compared to the viral strategy, the non-viral approach can easily be re-administrated thanks to the absence of an immunogenic reaction and to their good tolerance [15]. This re-administration is mandatory because of the constant renewal of epithelium pulmonary cells leading to the decrease in and eventually the disappearance of transgene expression. Moreover, non-viral vectors have the ability to compact longer nucleic acid sequences, which is useful for transgenes such as CFTR. However, the in vivo efficiency of non-viral vectors is still too low to get closer to the normal phenotype significantly, even if a change in the clinical state of the patient can be observed [15]. These strategies still have to be improved.

Given its characteristics, CF is a particularly complex and challenging model to approach pulmonary diseases and is a helpful model for other pathologies affecting the lungs and the respiratory tract. Successfully developing a gene therapy treatment for CF can potentially open the door to the treatment of other lung disorders. To directly target the lung, inhaled drugs can be administrated under different forms: pressurized metered-dose inhalers, nebulizers, soft mist inhalers, nasal sprays, and dry powders for inhalation (Figure 1A) [16,17]. The choice of devices has some consequences and influences the lung deposition [18]. This type of administration is a non-invasive method performed locally as drugs can easily reach the large absorptive surface area of the epithelium. Moreover, this technique has the benefit of avoiding the first-pass metabolism, intestinal efflux transporters, and degradation in the digestive system [19,20]. Deposition of an aerosol is dependent on the physical properties of generated micro-droplets. The droplet size determines the delivery location in pulmonary airways: the smaller the size, the deeper the deposition. The anatomy of the upper and lower airways and the breathing frequency are also key parameters that influence aerosol deposition [21,22]. Nebulization represents the process of transforming a liquid into an aerosol which can reach the respiratory tract (Figure 1B). This technique has the advantage of not requiring galenic formulation to a dry powder, which could damage the quality of the product. Qiu et al. showed that the lyophilization technique did not impact the in vitro transfection efficiency but, when tested on mice models using tracheal administration, nebulization of a reconstituted formulation was more efficient than the dry powder [23].

### 1.3. Current Challenges and Models

However, currently, over 25 clinical trials of gene delivery based on viral or non-viral gene vectors have failed to demonstrate significant clinical benefits, mainly due to inefficient gene transfer into the target cells [24]. Additionally, some viral CF gene therapy trials elicited host immune response. Consequently, clinical assays have been discontinuous, making subsequent treatments ineffective. Moreover, lifetime repeated treatment is required when using non-integrative systems for CF patients because of the transient nature of episomal transgene expression in addition to the natural life expectancy of the airways epithelial cells [25]. In 2015, the UK CF Gene Therapy Consortium completed a CF gene therapy clinical trial and observed that the use of a non-viral gene vector (GL67A, Genzyme corp.) had a significant, but still modest, benefit on the respiratory function (+3.7% of FEV1) compared to placebo controls [15]. They showed that a single aerosol per month, for one year, of a solution composed of nanoparticles (NPs) containing 13 mg of plasmid was very well tolerated. However, a more efficient gene delivery vector is needed to really make gene therapy a realistic option for the treatment of CF patients [15].

Extracellular barriers, indeed, have to be taken into account to adapt and finely tune their formulations (Figure 1B). Due to the presence of mucus as a protective barrier for xenobiotics, the passage through the cell is considerably reduced. The great majority of non-viral vectors as well as viral ones are stuck in the thick mucus of CF patients [26]. Inhaled gene vectors can be trapped within the mucus layer via non-covalent bonds (electrostatic and hydrophobic interactions) or simply cannot enter the network structure [26]. Many clinical studies have failed to demonstrate a gene therapy efficiency in the CF lung because the mucus was partially or totally neglected in in vivo and in vitro models. For instance, GL67A was selected using healthy sheep, and therefore without taking the mucus into account [27]. Moreover, animal models currently used for gene transfection studies are not totally satisfactory for CF model systems. For instance, CF mice and rats do not present spontaneous lung infections as well as mucus production [28]. W. K. O’Neal et al. successfully developed a transgenic mice model with overexpression of the ENaC β-subunit [29]. This model has the advantage of spontaneously exhibiting a CF-like lung disease more similar to human pathology with airways inflammation, bacterial infection, mucus dehydration, and obstruction [30,31]. Due to their anatomy, however, larger animal models represent better models to test inhaled gene therapy because of closer physiological and pathological similarities to human lungs. CFTR-knockout ferrets can be used as a potential model due to a lung structure close to that of humans. They exhibit altered chloride transport, leading to mucus hypersecretion and lung infections [32,33]. CF pigs also represent a potential model, as they present pulmonary manifestations similar to those found in human CF lung (infection, inflammation, airway remodeling, and mucus hypersecretion). They have, however, a short life expectancy (some days to few weeks) [34,35]. Nonetheless, every animal experimentation has to respect the 3R regulation (Reduce, Replace, and Refine). Difficulties in accessing animal models coupled with 3R restriction lead to the need for a more accurate cellular model. If the models used in vitro or in vivo are not sufficiently complex and representative of clinical conditions, the results obtained will not be reproducible during clinical trials. Therefore, it appears to be essential to have a reliable in vitro model close enough to the pathology studied to test and develop new vectorization strategies for nucleic acids with the ultimate aim of gene therapy. This review will focus on the use of mucus models and cellular cultures to obtain results closer to the in vivo pulmonary tracts and to avoid, as far as possible, failures in clinical development. Such models are fundamental to allow the development of non-viral gene carrier formulations able to overcome cellular and extracellular barriers faced in the respiratory tract.

## 2. Mucus Model for Gene Therapy

### 2.1. Mucus Composition and Biological Fuctions

#### 2.1.1. Airway Mucus

As previously mentioned, epithelial pulmonary cells are covered with airways surface liquid (ASL). The thickness of ASL has been estimated to be between 10 and 20 µm [36]. Two layers form this surface liquid: the periciliary layer (PCL) and the mucus layer. The PCL is the deepest film that covers the epithelial cilia and facilitates the beating. This layer is less viscous than the upper coat forming the mucus layer. Mucus is a hydrogel that can be found on the surface of wet epithelia such as the lungs but also the stomach, eyes, and vagina. It has a key role in protecting epithelial cells. Mucus is a dynamic semipermeable barrier that enables the exchange of nutrients, water, gases, odorants, and hormones, but retains pathogens and particles through its network structure [37]. Mucus is constitutively excreted by secretory cells in the airway and removed by MCC. Mucus combined with the mucociliary escalator of the upper airways allows the trapped particles to be moved back up to the airway and mainly swallowed into the esophagus [38]. The clearance of inhaled matter generally occurs in 15 min to 2 h and is dependent on several factors (anatomy, size and deposition of particles, tobacco exposure, and pulmonary disease) [39]. Along the lower airways, in the alveolar region, a surfactant film is present and participates in the oxygenation function. Pulmonary surfactant is a surface-active complex composed of lipids and proteins. This fluid prevents alveolar collapse at low lung volume. It also preserves bronchiolar patency during respiration while protecting against injuries, inhaled particles, and micro-organisms [40].

#### 2.1.2. Mucins

**Figure 2:**
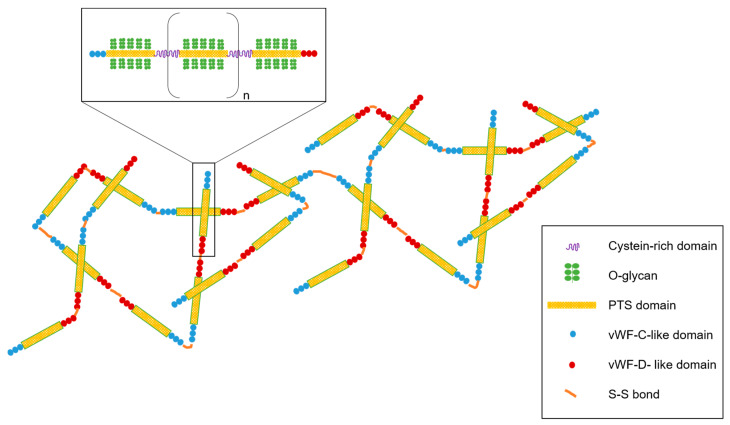
General structure of mucins, the main constituent of mucus. Mucin contains the PTS (proline, threonine, and serine) domain and supporting O-glycan. The PTS domain is interspaced with cysteine-rich domains. Mucin glycopolymer ends with the von Willebrand factor (vWF) domain in N- and C-terminal regions. Mucin polymerized via disulfide bonds and mucin polymers interact with each other via hydrophobic and electrostatic interactions.

In a healthy context, mucus is constantly maintained in a state of hydration thanks to the coordination of CFTR and ENaC channels. Mucus is mainly composed of 95% water, but one of the main constituents playing a key role in this physical barrier is the structural glycoprotein mucin (size 0.5–50 MDa) [41]. Mucin acts as a net that traps molecules which cannot cross the pores, whose size lies between 10 and 500 nm [41,42]. Two major classes of mucins exist: mucins that are secreted and mucins attached to the cytoplasmic membrane (MUC1, MUC3A, MUC3B, MUC4, MUC12, MUC13, MUC15, MUC16, MUC17, and MUC20) [43]. Membrane-tethered mucins possess transmembrane and cytoplasmic domains. Some of them also present an epidermal growth factor-like domain [44]. Among the secreted mucins, five oligomeric gel-forming mucins (MUC2, MUC5AC, MUC5B, MUC6, and MUC19) and two non-polymeric mucins (MUC7 and MUC8) form the structure of the protein backbone [43]. Secreted mucins are stored within secretory vesicles and, constitutively or in response to a stress, are released to protect the airway epithelium. The airway mucus is mainly composed of MUC5AC released from goblet cells and MUC5B produced by the mucous cells in submucosal glands [43]. MUC2, also present in the respiratory mucus, is found at the bronchus level at a low concentration [45]. Mucin secretion is mediated by different factors such as pathogenic agents and inflammatory mediators by increasing mucin gene expression [5,46]. Secreted or not, mucins share some common structures. The mucin central region is composed of a high content of serine, threonine, and proline residues (PTS domain), leading to a dense glycosylation (Figure 2) [47]. The glycosylated portion represents 70 to 80% of the total mass of mucin and is composed of carbohydrates such as N-acetylglucosamine, N-acetylgalactosamine, fucose, galactose, N-acetylneuraminic acid (sialic acid), and traces of mannose and sulfate [37,48]. Altogether, they form oligosaccharides linked by O-glycosylation that are responsible for the bottle-brush structure and for the global negative charge of mucin [47]. However, PTS domains are interrupted by cysteine residues playing a key role in the mucin network [49]. Moreover, amino-terminal and carboxy-terminal regions are rich in cysteine and create intradomain disulfide bonds (Figure 2). N- and C-terminals of the protein backbone also have some similarities with the sequence of von Willebrand factors C and D and are involved in polymerization of the mucin network [50]. In addition to disulfide bonds, hydrophobic and electrostatic interactions also contribute to the formation of a 3D network responsible for the gel-type characteristics of mucins [51].

### 2.2. Mucus Physical Properties

#### 2.2.1. Mucus Rheology

Rheology is the science of deformation and flow of matter. Rheological techniques are often used to investigate the relationships between the behavior of complex fluids and their multi-scale structure [52]. Since pioneer works in the 1960s, the rheological properties of mucus, which is a natural complex fluid, have attracted much attention [53]. Using classical macrorheology, the effect of mucus viscoelastic behavior on mucus transport properties was highlighted [54,55]. From a therapeutic aspect, the better understanding of the role played by rheology in mucus transport allowed improving mucus drainage [56,57]. More recently, the interest in using microrheology in order to investigate local viscoelastic properties of mucus was underlined [58]. Indeed, microrheology is more interesting to investigate the viscoelasticity that will encounter microparticles or nanoparticles. This technique can appreciate the heterogeneity inside the mucus samples and represents a necessary complement to macrorheology by characterizing local mechanical properties in biological fluids [58]. At last, there are numerous recent studies concerning the search for rheological properties as characteristic markers of respiratory diseases [59]. From that point of view, characteristics of the rheological response in a non-linear regime (large deformation or flow regime) seem to be better biomarkers than the viscoelastic response in the linear regime [60].

#### 2.2.2. Factors Influencing Mucus Rheology

Mucus is mainly composed of water and mucins. However, other molecules such as immunoglobulins, lipids, DNA, electrolytes, inorganic salts, enzymes, cells, cellular debris, and mucopolysaccharides take part in the mucus composition [61]. Taken together, the normal mucus gel barrier constituents create a heterogeneous environment and directly influence the Newtonian viscosity, which is 1000 to 10,000 times higher than that of water [41]. In diseases such as CF and chronic obstructive pulmonary disease (COPD), MUC5B is found to be predominant, whereas in healthy patients, MUC5AC is the main mucin [62]. These unbalanced situations lead to different rheological properties. Mucus exhibits viscoelastic behaviors which correlate with the hydration ratio and mucus composition. Moreover, vitamin A (acid retinoic) has also been shown to increase mucin gene expression [63,64]. Furthermore, in the CF and COPD context, some changes in glycosylation may occur, leading to an increase in sulfatation of mucins and contributing to phenotype degradation [65]. In these diseases, mucins are overproduced and the transport properties of nanoparticles onto the epithelial cells are very limited, and, instead of protection, mucus provides a propitious environment for bacteria development. The emblematic pathogen infection in the CF patient is *Pseudomonas aeruginosa* (PA). PA is a Gram-negative opportunistic bacterium with flagella, which is capable of biofilm formation in the airway respiratory tract. When PA aggregates, it loses its mobility and adopts anaerobic respiration, avoiding macrophage destruction [66,67]. Moreover, this bacterium can be hooked to the epithelial via adhesion or to the mucus layer thanks to its flagella that have the ability to bind to MUC1 [68]. Bacterial infections lead to chronic inflammation and pulmonary damage. Infection and the inflammatory environment have also been shown to change mucin glycosylation. In chronic respiratory diseases, mucin production increases due to inflammation which recruits and activates T cells and macrophages, but also neutrophils and eosinophils [5]. For example, Delmotte et al. pointed out the fact that the cytokine tumor necrosis factor (TNFα) has the faculty to alternate sialylation in tracheal cells [69]. Some studies have demonstrated that neutrophil elastase and interleukin-9 increased the expression of MUC5AC in epithelial pulmonary cells [44,70]. Pro-inflammatory cytokines such TNFα, IL-6, and IL-8 directly influence the rheological properties of mucus. Moreover, in the CF airway, a high level of DNA and actin filaments is observed due to the necrotic neutrophils present in this pathology. They contribute to the dense network structure of the mucus. For instance, DNA makes up about 0.02% of mucus mass and is originated from debris of epithelial cells [71]. However, in CF patients, there is an accumulation of DNA, which increases both the elasticity and viscosity of the mucus [72]. This DNA results from the destruction of neutrophils and its mass ratio in mucus can rise up to 1.5% [73]. A commonly used mucolytic for CF sputa is rhDNAse (Pulmozyme^®^) that directly targets and hydrolyzes the extracellular DNA. Lipids are also present in the mucus, with a mass ratio up to 1–2% [71]. In healthy mucus, pH is around 7; however, in CF, because of the bacterial development, pH is around 5 [74]. This variation also influences the physical properties of the mucus barrier and its viscosity and impedes the nanoparticle diffusion. Lipids are also mainly found in hydrophobic domains of mucin and play a crucial role in the mucus viscosity [75]. For instance, phosphatidylethanolamine, sphingomyelin, and lysophosphatidyl-choline increase the viscosity, whereas phosphatidylglycerol decreases the CF mucus viscosity [76,77]. Finally, mineral salts present in mucus influence the ionic strength and consequently the structural and rheological properties of mucus.

### 2.3. Mucus Sources

To determine the capacity of gene vectors to overcome the mucus barrier, mucus models used in vitro should possess physical and biological properties similar to those of native mucus (Table 1). Purified mucin is commercially available, but in 2010, Crater and Carrier showed that gel reconstituted from purified gastric mucin (PGM, Sigma-Aldrich) did not represent an ideal model [78]. They concluded that this model was not accurate to study particle diffusion into the mucus. Moreover, using rheological techniques, Kočevar-Nared et al. compared crude gastric mucin and natural gastric mucus and pointed out the fact that they had different rheological behaviors, due to purified mucins which did not form a proper network [51]. The extraction protocol for mucin isolation perturbs the interaction involved in the supramolecular organization. When disulfide bonds are disrupted by chemical reduction, the gel collapses. Mucin purification alters native interactions but also interactions between the other mucosal compounds. This disruptive condition leads to a poor rheological model for studying the physical behavior of the airway mucus. More recently, Meldrum et al. showed that the mucin gel assembly is formed by a synergistic interaction between mucin, but also non-mucin proteins, and Ca^2+^ [79]. Artificial models have also been used to study nanovector interaction. D. Sriramulu and coworkers developed an artificial sputum medium (ASM) to mimic the sputum of CF patients [80]. To study microparticles of mannitol containing ciprofloxacin, Yang et al. used this ASM model [81]. These microparticles were proved to be able to penetrate artificial mucus, but this ability remains to be demonstrated on human mucus. Freshly collected mucus should ideally be used for such studies. The mucus origin and the species are also important. Ideally, human mucus would be used. The physical and biological properties of gastric mucus are different from vaginal mucus as well as airways mucus. For instance, under a non-pathological condition, the pH of gastric and vaginal mucus is acidic, whereas pulmonary mucus is neutral [47]. Human airway samples can be collected by an endotracheal tube, brushed or directly collected from patients’ expectoration. To really assess the mucus properties and determine if nanoparticles are capable of crossing over, the storage of samples has to be specific and carefully controlled. When the mucus is kept at 37 °C up to 60 min, proteases will degrade mucins. Frozen mucus (between −20 and −80 °C), due to the reduction in protease activities, retains its bulk viscoelastic properties. However, when sputum is kept at 4 °C for more than 48 h, the diffusion of NPs decreases [26].

### 2.4. Crossing the Mucus Barrier

Gene vectors that have been tested for the CF treatment in clinical trials have not been proved to efficiently penetrate the mucus barrier, even for viral vectors [90]. Non-viral vectors, mostly cationic, are also unable to pass through the mucus network mesh due to their size and their electrostatic interactions. Cationic gene vectors aggregate in the presence of albumin that is abundant in the airway secretions [91]. Aggregation can also be observed with the DNA and mucin glycoprotein present in the mucus, especially in CF patients. Moreover, the size of the nanoparticles is primordial for the penetration into the mucus even in healthy donors [92]. They have to be small enough to get through the mesh of the network structure. Airway mucus pores are about 500 nm large; however, for CF mucus, the pore size is smaller, approximatively 150 nm, meaning that NPs also have to be smaller [93]. Murgia et al. also showed that the penetration of nanoparticles into porcine respiratory mucus after aerosol deposition is size-limited [82]. After aerosol deposition on mucus, NPs of 200 nm and larger could not penetrate the mucus. However, a small size is not enough to ensure the rapid transport of NPs in mucus. Nanoparticles (NPs) surface chemistry represents an important factor influencing the capacity to overcome the mucus barrier. There are different strategies to break through the mucus barrier. Uncharged NPs are needed to avoid electrostatic interactions with the negatively charged mucins. For example, the use of poly-ethylene-glycol (PEG) covalently attached on the NPs surface has been shown to reduce the electrostatic interactions between the positively charged nanoparticles and the negatively charged mucus [94]. Suk et al., in 2008, showed that non-adhesive NPs (PEG-PS) of 100 nm were more hindered in CF sputum than 200 nm PEG-PS, but less hindered than 500 nm PEG-PS [42]. The authors explained the results by the insufficient PEG coating of 100 nm PEG-PS, thus underlying the importance of the chemistry surface. PEG coating techniques also need to be considered. NPs densely coated with linear PEG have been shown to efficiently pass the mucus barrier. Xu et al. demonstrated that PEG with a low density (mushroom conformation) did not sufficiently cross mucus and interacted with mucin. On the other hand, PEG with a high density (dense brush conformation) largely diffuses in mucus [95]. Mansfield et al. tried to replace PEG with poly(2-oxazoline) (POZ) and reported that silica NPs with POZ enhanced diffusion into mucin [96]. Another option to coat NPs is the use of triblock copolymers of poly(propylene oxide) and poly(ethylene oxide) (PEO-PPO-PEO) [97]. In this case, the triblock copolymers, namely, Pluronics, were physically adsorbed on the surface. They showed that Pluronic F127, which is the most commonly used Pluronic, improved the transport of polymeric particles. It was also shown that the hydrophobic part, PPO, can be associated with the hydrophobic NPs surface, whereas PEO avoids muco-adhesion. Instead of fixed Pluronic F127 in the NP surface, Ensign et al. pretreated cervico-vaginal mucus with it [98]. They reported that F127 improved the distribution, retention, and efficiency of nanoparticles. The use of the HPMA (N-(2-hydroxypropyl) methacrylamide) copolymer was also explored as an alternative to PEG. In parallel, Shan et al. designed insulin-loaded NPs coated with HPMA that allowed penetration of the mucus and released insulin inside epithelial cells [99]. Alternative strategies focus on the mucus mesh perturbation by using mucolytic agents. Two main mucolytic approaches exist: the use of disulfide-breaking molecules and the use of proteolytic enzymes. In each case, the mucus structure is disrupted, enabling the penetration of nanoparticles. Dithiothreithol (DTT) is commonly known to cut disulfide bridges but is also cytotoxic. As a consequence, it cannot be used in clinical applications but remains a valuable control in vitro. Another agent that can be used to reduce disulfide interactions is N-acetyl cysteine (NAC), which has one sulfhydryl group that allows decreasing the mucus viscosity. Suk et al. proved that the pretreatment of CF sputum with NAC enhanced rapid transport of muco-nanoparticles [100]. Based on PEGylated nanoparticles, they showed that the average size of the sputum mesh increased from 145 ± 50 to 230 ± 50 nm when pretreated with NAC. Developing nanoparticles releasing mucolytic agents therefore represents a good strategy to allow nanoparticles to find their way inside the mucus structure. Proteolytic enzymes, such as trypsin, papain, and bromelain, were immobilized on nanoparticle polymers to study their ability to digest the glycoprotein network and disulfide bridges [101]. The authors used this particle on intestinal mucus and noticed that such nanoparticles could penetrate the mucus.

## 3. Cellular Models for Gene Transfer Evaluation

### 3.1. Respiratory Tract

The use of relevant in vitro models is essential to mimic the in vivo situation and properly evaluate the efficiency of innovative treatments (Table 2). In fact, cellular models can be a valuable alternative to in vivo efficiency studies, as they can be highly reproducible. The airway pulmonary tract constitutes a major organ due to its function and represents the location where gas exchanges occur in mammals. This system can be roughly subdivided into two parts: the upper and the lower respiratory tracts (Figure 1A). The upper part is composed of nasal cavities, the pharynx, and the larynx, while the lower tract includes trachea, bronchi, bronchioles, alveolar ducts, and alveolar sacs [102]. The proximal part, from nasal cavities to the second or the third segmentations of bronchioles, is a cartilaginous airway, while the distal airway is non-cartilaginous, corresponding to the terminal and respiratory bronchioles [103]. Different types of cells, constituting the pulmonary pseudostratified epithelial, can be found in the pulmonary tract. All these cells play a direct or indirect role in the oxygenation function. Between 6 and 12 L of air are inhaled every minute and constantly expose the lungs to microorganisms, allergens, and particles (sized nm to µm). To prevent such aggression, the lungs have to possess different barriers to contain any kind of particle. The integrity of the epithelium combined with the ASL presence is essential to prevent potential pathogens from penetrating the bloodstream [104].

### 3.2. Airway Epithelium

**Figure 3:**
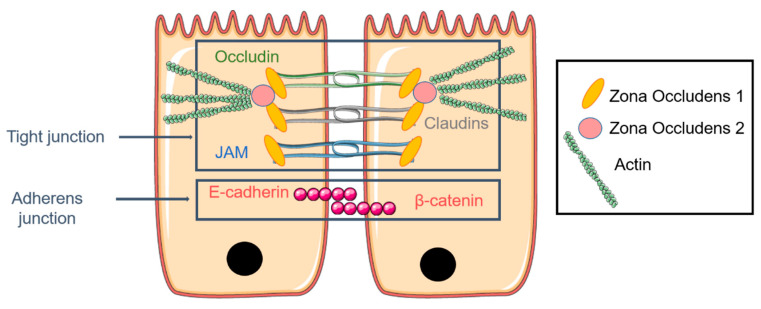
The pulmonary epithelium is maintained by adherent junctions and the tight junction (TJ) complex composed of occludin, claudins, and junction adhesion molecules (JAM). Zona occludens (ZO) is directly attached to actin and connects the TJ to the cytoskeleton, notably actin F.

The airway epithelium is mainly composed of ciliated and secretory cells contributing to the protection and humidification of the pulmonary mucosa. The epithelium of the respiratory mucosa is a physical barrier maintained by junctions between cells. These junctions are composed of occluding tight junctions (TJ), anchoring adherent junctions, desmosomes, and Gap junctions (Figure 3) [105]. TJ formation appears during the polarization of epithelial cells. This process is led by the sorting and the distribution of certain protein and lipid membranes which organize the cell in three surface domains (apical, basal, lateral) [106]. TJ complexes are made by occluding, claudins, and junction adhesion molecules (JAM). A TJ complex consists of a TJ part and an adherence junction mainly composed of E-cadherin [107,108]. The selection of cell type to model the airway epithelium requires, in fact, the formation of functional TJ by cells in culture when cultured in air–liquid interphase (ALI) conditions (see ALI and TEER measurement paragraph) [109]. These properties can be estimated through the measurement of the transepithelial electrical resistance value (TEER expressed in Ω·cm^2^). This technique allows the determination of the integrity of TJ in culture of epithelial cells through the use of electrodes [110]. For instance, tracheal and bronchial epithelial cells culture can be used as a model due to the cells’ ability to differentiate and to provide a permeability barrier. The pulmonary epithelium is also a polarized pseudostratified epithelium which is composed of three major kinds of cells with distinct properties. Among them, ciliated cells represent over 50% of all epithelial cells [111]. In the upper airway, the predominant cells are ciliated columnar cells interspersed with goblet cells, while cuboidal ciliated cells interspersed with club cells are present in the lower airway [5]. In the epithelial airway, there is approximately one secretory cell for five ciliated cells; this ratio increases in diseases such COPD, asthma, or CF [5,104].

#### 3.2.1. Ciliated Cells

Ciliated cells are elongated columnar cells that contain abundant mitochondria in the apical region to ensure the production of ATP, mandatory for the ciliary motion [103]. About 200 to 300 cilia recover the apical pole of each ciliated cell [104]. Airway cilia are 6.5 to 7 µm long, with a diameter of 0.1 µm, and are structured by microtubules [112,113]. The main role played by these cells is to propel the mucus layer produced by the secretory cells. Coordinated beatings take place to sweep the mucus and everything trapped in it from the luminal space towards the pharynx where it is most often swallowed. The ciliary beating frequency (CBF) is approximatively 12–14 Hz for cells cultivated in vitro. The CBF is dependent on different kinds of stimuli [114]. For instance, it has been shown that Ca^2+^ plays a key role in the CBF regulation. When [Ca^2+^] is increased in mammalian cells, CBF is increased, and vice versa [115,116]. The composition of the ASL directly influences the mucociliary clearance (MCC), especially for diseases such as CF where dehydrated mucus sticks to cilia and prevents them from expelling the mucus by reducing their motion.

#### 3.2.2. Secretory Cells

The principal secretory cells present on the airway epithelia are the goblet cells which are intercalated between ciliated cells. Goblet cells are highly polarized cells. Nucleus and organelles are observed at the basal pole, whereas membrane-bound secretory granules of mucin are present at the apical pole, giving the cell its characteristic shape [103]. Secretion is mediated by a secretory apparatus engaged in mucin granules exocytosis. This phenome is ATP-dependent and involves the P2Y2 receptor (G protein-coupled receptors) [93]. When ATP is attached on this apical membrane receptor, phospholipase C (PLC) is activated, which triggers the second messengers: diacylglycerol (DAG) and inositol triphosphate (IP3). DAG activates Munc13 that liberates Syntaxin and therefore the formation of a four-helix SNARE (soluble N-ethylmaleimide–sensitive factor attachment protein receptor). When complexed with SNAP-23 (synaptosomal-associated protein 23) and VAMP (vesicle-associated membrane protein), the membrane of the secretory vesicle is allowed to be closer to the plasma membrane. As for IP3, attachment to its receptor enables the release of intracellular calcium from the endoplasmic reticulum. This calcium increase induces the activation of synaptotagmin, leading to the final confirmation of the SNARE complex [117]. All these events lead to the fusion of both vesicle and plasmatic membranes and to the release of mucins [118]. Submucosal glands constitutively secrete mucins, but its production can be increased through adrenergic and cholinergic stimulation [119]. In the pathologic context, the number or size of the cells is disturbed and hyperplasia in goblet cell occurs in COPD and asthma, while hypertrophy concerns CF patients [120]. Other kinds of secretory cells can be found in the small airways, namely, serous and club cells, which also participate in the humidification of epithelial cells. Club cells also have the ability to differentiate into ciliated cells or goblet cells [103].

#### 3.2.3. Other Cell Types

Another kind of cell called basal cells are progenitor cells, linked to the basement membrane through hemi-desmosomes; they interact with columnar cells as well as neuronal cells. In addition to their ability to repopulate the epithelium cells of the conductive airway, they can also regulate inflammation response, water movement, or oxidant defense [121]. Apart from these tissue-specific epithelial cells, there are a variety of non-epithelial cells, such as immune cells (lymphocytes, leukocytes, and mast cells), and squamous alveolar type I pneumocytes covering 96% of the alveolar region, which are responsible for the gas exchange, or cuboidal alveolar type II pneumocytes involved in the synthesis and secretion of antimicrobial and surface-active components [122,123,124]. More recently, a new kind of cell, ionocytes, has been identified; they have been described as a major source of CFTR activity [125,126]. Although these cells represent only 1% to 2% of the total cells of the pulmonary epithelium, they accumulate more than 50% of *cftr* transcripts and could represent an important target for CF gene therapy.

### 3.3. Immortalized Cells

Immortalized cells have undergone genetic modifications leading to an indefinite replication. They can be either native from a spontaneous tumor or can be transformed by oncogenic or viral agents. Among airways epithelial cells, these kinds of cells are of particular interest because they proved to be useful in screening new drugs and are available in bulk quantities. Immortalized epithelial cells from airways were established by the introduction of exogenous DNA in order to provide better characteristics of the relationship between transmembrane conductance regulation and CF pathology [127]. Different strategies were tested to immortalize cell lines, the most used and particularly efficient one is the introduction of DNA encoding the SV40 T-antigen. The development of this cell line allows high-throughput screening to identify new drugs as well as genetic and pharmacological therapies. However, these systems present some disadvantages, especially for in situ extrapolation [128]. For instance, the clone is from a mixture of primary epithelial cells with different phenotypes; therefore, an isolated clone cannot represent all the characteristics of the original tissue. Furthermore, when the cells are kept in culture, they can express different properties than their original features. The transformation itself also influences the phenotype expression. As an example, they can lose their ability to form TJ, ciliary beating, mucus secretion, and their properties to transport ions, depending on the cell lines [129].

#### 3.3.1. BEAS-2B, NuLi-1, NCI-H292, NCI-H358

BEAS-2B is a normal human epithelial cell transformed by an adenovirus 12 and SV40 hybrid [130]. This cell line is commonly used for studying airway epithelial structure and function and the interaction between xenobiotics and the cells themselves [131]. NuLi-1 is an airway epithelia cell which is useful for studying passive and active drug transportation through the epithelial barrier [132]. NuLi-1 cells have the ability to form TJ with a TEER value around 685 ± 31 Ω and can be used for active and passive drug transport studies [132]. NCI-H292 is a cell line obtained from a 32-year-old woman with mucoepidermoid pulmonary carcinoma. They do not form tight junctions but express desmosomes and adherens junctions. They also present a low level of CFTR, while they express mucin and can be used for mucin expression studies [133]. NCI-H358 cells were isolated from a man with a bronchioalveolar carcinoma and have the ability to express lung surfactant associated protein A (SP-A) [134].

#### 3.3.2. 16HBE14o- and CFBE41o-

**Figure 4:**
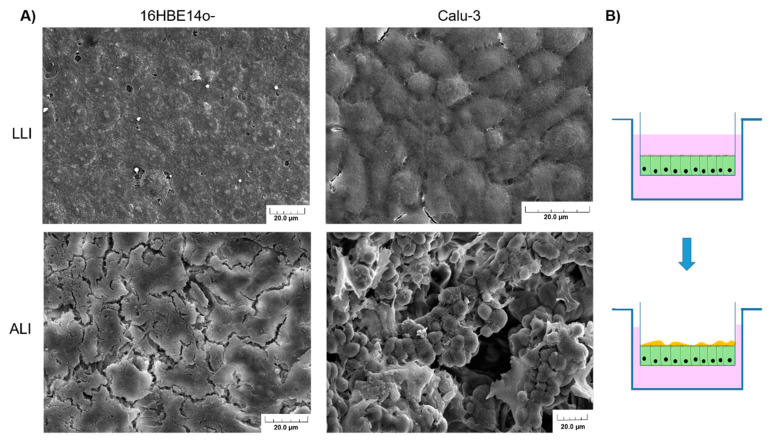
(**A**) Scanning electron microscopy of 16HBE41o- and Calu-3 cultivated in the liquid–liquid interphase (LLI) or air–liquid interphase (ALI) for 3 weeks after seeding. When cultivated under ALI conditions, unlike 16HBE41o-, Calu-3 cells produce mucus and form microvilli. For all pictures, the scale bar corresponds to 20.0 µm. (**B**) Transition from LLI culture to ALI allowing cell differentiation and in some case, depending on the cell line, mucus production.

The 16HBE14o- cell line was developed by Cozens et al. It exposes a transepithelial resistance at 250 Ω·cm^2^ after 6 days of optimized culture depending on initial seeding densities and surface coating [135,136]. This cell line expressed more P-glycoproteins, lung resistance-related proteins (LRP), and caveolin-1 [137]. 16HBEo- was initially developed for the study of CFTR but can also be used for transport studies. Under ALI conditions, they do not polarize and do not display a normal epithelial phenotype (Figure 4A). Another cell line, the immortalized CF tracheo-bronchial epithelial cell CFBE41o-, is homozygous for ∆F508-CFTR and was first used by D. Gruenert and his team [138]. Ehrhardt et al. reported that CFBE41o- had a TEER peak at 1156 Ω·cm^2^ after 16 days of liquid-covered culture, while TEER is around 200 Ω·cm^2^ for cells cultured under ALI conditions [139]. This cell line is particularly interesting to study gene transfection or drugs screening in the CF context, due to its homozygous state.

#### 3.3.3. A549

The A549 cell line is a human lung adenocarcinoma from a Caucasian male with lung cancer [140]. This cell line is frequently used as an in vitro model of pulmonary cuboidal epithelium or alveolar cells and screening of biopharmaceuticals molecules. For example, this cell line can be used for transfection assays, drug testing, and infection studies. However, A549 does not form TJ when cultured in ALI conditions. This feature leads to the formation of a very permeable monolayer with low transepithelial electrical resistances. In some cases and in optimized co-culture conditions, TEER values of this cell line can be increased [141]. Moreover, A549 possesses several multilamellar cytoplasmic inclusion bodies that are reminiscent of alveolar cells and can also be used as an alveolar model [142].

#### 3.3.4. Calu-3

Calu-3 cells are cells from a 25-year-old Caucasian male with lung adenocarcinoma. When cultured in ALI, they formed a pseudostratified columnar epithelium similar to the normal columnar bronchial epithelium [143]. In such culture conditions, Calu-3 presents far more secretory vesicles after 3 weeks and mucus secretion can be observed. This secretion is also due to the fact that Calu-3 can differentiate into goblet cells. From our own observations, scanning electron microscopy reveals the presence of abundant microvilli in the apical surface (Figure 4A). Moreover, Calu-3 cells form a confluent monolayer and express TJ protein occludin, adherens junction protein E-cadherin, and epithelial-specific cytokeratin 7 (CK7) [144]. Calu-3, in ALI conditions, presents a morphology very close to the in vivo airway epithelium in terms of ultrastructure, secretory component, and electrical resistance [145,146]. The TEER values of Calu-3 in ALI culture show a variable TEER, ranging approximately between 400 and 800 Ω cm^2^, but confirming the formation of a tight epithelium [109,147]. A previous study has shown that Calu-3 could be transfected by polyethylenimine (l-PEI) and therefore could be used as a potential model for gene delivery [148]. Calu-3 is close to the in vivo situation, as it secrets mucus and generates microvilli. For instance, Calu-3 can be used as a good reproducible aerosol model for gene delivery. Hein et al. developed the new Pharmaceutical Aerosol Deposition Device on Cell Cultures (PADDOCC) and used the Calu-3 cell line to mimic the inhalation of dry powder on an epithelial monolayer [149]. Furthermore, Jeong et al. very recently proposed a system using the Calu-3 epithelium, able to reproduce the biology of the respiratory tract when it is exposed to chemicals [150].

### 3.4. Primary Cell Culture

Immortalized cell lines have low culture cost and provide large amounts of reproducible cells, yet despite their advantages, they frequently lose their characteristics during in vitro culture and senesce after a certain number of divisions. In addition, they are not a perfect representation of the original cells in primary culture; this is especially true for cancerous cells which are morphologically different [137]. Primary cell cultures, on the other hand, represent a closer model to the in vivo situation than immortalized cells, due to the fact that they are directly isolated from animal or human tissue and used as they are. However, cell isolation is hardly reproducible and collected cells are heterogeneous, meaning that each isolation is somewhat unique. There is also the non-negligible risk of bacteria and fungi contamination, as well as a limited life expectancy compared to immortalized cell lines.

#### 3.4.1. Mammalians Cells

Primary epithelial cells can be derived from the upper and the lower airways. These cells can be collected from different species such as mouse, pig, bovine, or sheep. Lam et al. characterized a porcine bronchial epithelial cell culture model [151]. They took bronchi from the slaughterhouse and, after a dedicated protocol of digestion and cell culture, obtained a valuable differentiated airway epithelium. Moreover, Boyle et al. used trachea picked from sheep and cultivated on an air–liquid interphase. They observed the formation of ciliated epithelial cells from day 12, as well as mucus production [152]. TEER values were measured to have a peak at 1049 Ω·cm^2^ and stabilized around 200 Ω·cm^2^. Mouse trachea can also be used for airway epithelial cell isolation. Lam et al. thoroughly described the procedure to isolate mouse respiratory epithelial cells [153]. This model has demonstrated a TEER value between 1000 to 2000 Ω·cm^2^ and has been, thereafter, exposed to mainstream cigarette smoke. The formed epithelium exhibited several subtypes of cells: ciliated cells, basal cells, and non-ciliated cells highlighted by transmission electron microscopy observations. Cozen et al. characterized bovine bronchial epithelial cells (BBECs) when cultivated in ALI and proved that this model exhibited TJ, cilia, and mucus production [154].

#### 3.4.2. Human Cells

Human primary cells are the most relevant model because they are closest to the in vivo situation but remain difficult to access. The trachea and lung can be collected during organ transplantation, biopsies, excised human nasal polyps, or autopsies, reducing their accessibility considerably. Non-traumatic methods can also be used, such as cellular scraping or brushings, but the donor’s agreement is required. A primary cell culture is composed of different kinds of cells, including ciliated and goblet cells, which all contribute to mimic the in vivo airway. NHBE (Normal Human Bronchial Epithelial) is an in vitro model for human bronchial epithelial cells that has the property of forming functional barriers. When cultured in ALI conditions, NHBE presents peak TEER values at 800 Ω·cm^2^ after 8 days of cultivation [155]. The small airway epithelial cells (SAECs) model is a normal human lung tissue from the bronchiole area. It exhibits basal cell properties with expression of ICAM-1 (Intercellular Adhesion Molecule 1) and club cell specific protein (CCSP) [156]. This model has already been used for infection and co-infection studies but also for the study of nanoparticles and cigarette smoke exposure [156,157]. In CF, a pharmaceutical company, Vertex, has successfully used human primary cells isolated from the bronchi of a patient with G551D and F508del CFTR mutations to determine the efficiency of Ivacaftor and Lumacaftor drugs [158]. In parallel, Epithelix has developed a commercial model, MucilAir^TM^, which is a reconstituted epithelium using human primary cells issued from a single donor or a pool of donors. Cells could come from different anatomical sites (nasal, trachea, or bronchial) and could belong to healthy donors or patients. Several pathologies are available, such as COPD, asthma, or CF. It has been shown that these cells present a cilia-beating in ALI conditions with, amongst others, the formation of TJ, the production of mucus, and active ions transport. For instance, this model was used by Ishikawa et al. to study the direct aerosol exposure of cigarette smoke [159]. Moreover, Firth et al. used induced pluripotent stem cells (iPSC) from homozygote F508del patients as a model to test the CRISPR/Cas9 system in order to correct the mutation [160]. Vaidynathan et al. also used this technique on primary cells from patients to correct the mutation and succeeded in regenerating a well-differentiated epithelium on a porcine small intestinal submucosal membrane [161]. Conversely, the use of the CRISPR/Cas9 technique can generate new cellular models to study, for example, the influence of mutation on cell phenotype.

### 3.5. Methods of Culture

#### 3.5.1. Air–Liquid Interphase and TEER Measurement

**Figure 5:**
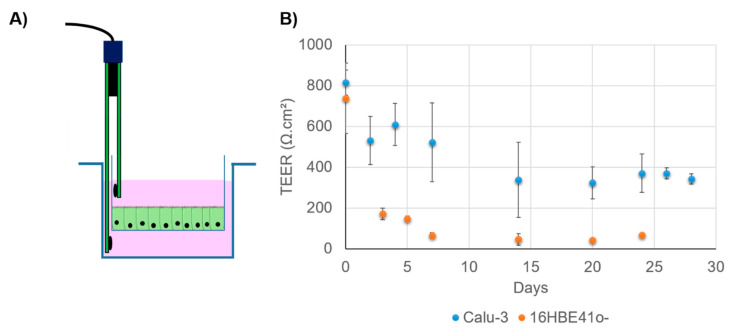
(**A**) Transepithelial electrical resistance (TEER) measurement using a Transwell and an electrode. (**B**) Evolution of TEER in Calu-3 and 16HBE41o- when cultivated in ALI conditions. Cells are seeded in a Transwell (150,000 cells per insert) and cultivated in LLI conditions for one week. Then, the apical media are removed. TEER is monitored for 25 to 30 days. Data are averages of at least three Transwells and are expressed in Ω·cm^2^. Calu-3 exhibits a higher TEER value, demonstrating its ability to form TJ under this condition.

Air–liquid interphase is a technique which can be used to mimic the respiratory tract epithelium (Figure 4B). This kind of culture is obtained by seeding airway epithelial cells (primary or not) in a Transwell system. After a proliferation phase, during which TEER is monitored, the media in the apical surface in the Transwell are removed to allow the cells differentiation into a pseudostratified epithelium. All nutrients, required to maintain the cells, are present in the apical chamber containing the culture media. Typically, in order to obtain a well-differentiated epithelial monolayer, cells have to be kept in ALI conditions for at least 3 weeks [143]. Primary cells, as well as immortalized cells, can be cultivated in such conditions. For instance, Calu-3 cells have the ability to differentiate under ALI conditions, forming cilia and producing mucus. NHBE also demonstrates such ability. This culture mode also presents the advantage of making a more complex model by adding mucus (artificial or from patient) on the apical pole of the cells that does not produce mucus spontaneously. To monitor the integrity of epithelium forms, TEER measurements can be used as previously mentioned (Figure 5A). They measure the electrical resistance across the cellular monolayer. This technique is very sensitive and informs researchers about the permeability of the epithelium [110]. The measurement method is based on Ohm’s law and reflects the ionic conductance. For the airway cells, it has been established that human tracheal and bronchial epithelial cells from healthy donors present a TEER value between 700 and 1200 Ω·cm^2^ when cultured in ALI conditions [162]. Pezzulo et al. also showed that the transcriptional profile of human primary ALI culture cells is closely similar to the native in vivo epithelium, demonstrating that ALI culture is more representative than immerged culture [162]. However, different TEER values have been reported for the same kind of cells, depending on the measurement protocol (Figure 5B). For instance, Foster et al. measured TEER values between 300 and 600 Ω·cm^2^ for Calu-3 cells, while Mathias et al. reported a TEER value for the same cell line at 1126 ± 222 Ω·cm^2^ [163,164]. It is important to note that in this study, Calu-3 was cultivated on a modified surface in the presence of collagen. In fact, there are numerous parameters to consider in TEER measurements: temperature, culture medium composition, cell culture passage, and shear stress [110].

#### 3.5.2. Culture Media

Culture media play a key role in the phenotype of ALI-cultured cells. For instance, primary cells, such as mouse trachea epithelial cells, when supplemented with IL-13, have the ability to produce mucus [165]. This interleukin up-regulates the production of mucus by inducing the production of MUC5AC [166,167]. Furthermore, cells (primary or not) can differentiate when cultured on collagen and supplemented with retinoic acid (RA), meaning that growth factors are important factors to be considered [168,169]. In the respiratory system, RA is the natural metabolite of retinol (vitamin A), controlling the development and the maintenance of the mucociliary epithelium [170]. Koo et al. showed that the incubation of RA on airway epithelial cells under squamous differentiation restored a mucous phenotype, leading to a mucin secretion 72 h after treatment [171]. Culture media can also influence ciliary motility. Compounds such as phenylmercuric borate and thiomersal have the ability to irreversibly decrease the CBF frequency. Conversely, chlorobutanol can inhibit motility with a reversible action. Another compound, benzalkonuim chloride, induces a slow, but irreversible, ciliotoxic effect. It also has the capacity to slow down CBF and disorganize the mucus structure through electrostatic interactions with anionic substances [172]. Mucociliary clearance constituted one of the extracellular barriers for gene transfer. Sinn et al. used formulations containing a viral vector with viscoelastic gels in order to slow down mucociliary clearance. This strategy was shown to considerably improve vector transduction [173].

#### 3.5.3. Polyculture

The pulmonary tract is composed of a multitude of airway epithelial cells and constitutes a complex tissue. To mimic the in vivo context as far as possible, polycultures have been considered. Altogether, cells create a particular airway microenvironment contributing to the pulmonary function and its protection. When mimicking, in vitro, the respiratory epithelium, polycultures represent a valuable approach and can bring results close to in vivo conditions. For instance, Rothen-Rutishauser et al. developed three lung cell lines associating A549 cultured with airway macrophages and dendritic cells to study particles interactions [141]. The use of a 3D model instead of a 2D model allows a mostly in vivo-like morphology, allowing a greater resemblance to the physiological conditions. Three-dimensional single or co-culture models also constitute an interesting system for in vitro studies. To obtain such models, the extracellular matrix is necessary to support cells. For that purpose, synthetic or natural hydrogels can be used and provide a valuable extracellular matrix [174,175]. Other kinds of support can be used as a scaffold such as porous sheets to mimic basal lamina. As an example, Harrington et al. used electrospun fibers of PET in order to create a 3D model of the airway epithelium [176]. Meenach et al. developed a lung composed of multicellular spheroids by using type I rat tail collagen with A549 and H358 cell lines. Two techniques were used in this study, liquid-covered culture and air–liquid interphase. These spheroids were then evaluated for aerosolization of dry powder to model lung cancer therapy [177]. Moreover, with the development of microfluidic technologies, a new approach of culture cells has been described, namely, the organ-on-chip approach. This in vitro approach allows high-resolution, real-time imaging analysis of living cells (structure and function) in a tissue or at the organ level [178]. The lung-on-chip model could become a relevant model for pulmonary drug delivery and especially for nanotoxicology studies because it can mimic the effect of lung–particle integration [142]. In fact, cells still form a tight epithelium barrier under this culture condition. Finally, an alternative approach is to use directly living tissue from the lung, trachea, or bronchus for constituting a relevant in vitro model. Different kinds of explant origins can be used such as human or porcine [179,180]. In 1994, Seeger et al. developed a model system using isolated and perfused rabbit lung [181]. This ex vivo model was afterward used as a pulmonary drug delivery for aerosolizable nanoparticles [182].

## 4. Conclusions and Outlooks

In vitro cell culture models of human airways are crucial complementary tools to properly study nucleic acid delivery under normal or pathophysiological conditions. As reviewed herein, gene delivery systems have to go through successive steps during their journey into the respiratory tract, which includes crossing the mucus barrier, reaching the underlying epithelium, and releasing their cargo in the cytoplasm of target cells. In this respect, CF may be considered as a challenging model disease since these steps are further complicated with noticeable hurdles, including mucus dehydration and accumulation, chronic inflammation, and microbial colonization. Importantly, improvements obtained for CF aerosol-mediated gene therapy could benefit other similar respiratory disorders such as asthma, chronic obstructive pulmonary disease (COPD), or α-1 antitrypsin (AAT) deficiency. Asthma is a chronic inflammation affecting the lower respiratory tract and characterized by difficulties to breathe due to reversible airflow obstruction and bronchospasms. COPD is a slowly progressive chronic disease mainly affecting bronchial tubes and alveoli and also characterized by a chronic airway obstruction that is not completely reversible with drugs [183]. Another genetic disease, α-1 antitrypsin deficiency, is caused by a missense mutation in the AAT-encoding gene involved in inflammatory processes. Finally, aerosol gene therapy may also be applied for treating lung cancers and metastasis located in the airways. Though different therapeutic nucleic acids may be used for each of the abovementioned conditions (e.g., miRNA, siRNA, or suicide gene), delivery systems and strategies will be needed in every case, which could be more properly designed thanks to optimized in vitro airway cell culture systems.

## Figures and Tables

**Table 1:** Example of different sources of mucus and their oscillatory shear viscoelastic properties. ω, angular frequency (rad/s); f, frequency (Hz); G’, storage modulus (Pa); G’’, loss modulus (Pa); η*, complex viscosity.

Species	Mucus	Method	Pathology	ω	f	G’	G’’	η*	Geometry	Ref
Pig	Airway	Collected from tracheas and stored at −4 °C	Non-pathologic		0.01–2	1–4	0.7–3.5		Cone-plate	[82]
	Intestinal	Mucin purified	Non-pathologic	10–100		0.3–10			Plate-plate	[79]
Human	Sputum	Transported on ice and directly analyzed	CF	0.1–40		30–100	20–30		Cone-plate	[83]
		Transported on ice and directly analyzed	CF		0.01–80	20–105		600–0.3	Cone-plate	[84]
		Stored at −80 °C	COPD		0.01–10	2–8		3–1	Plate-plate	[85]
		Sputum induction (4.5% hypertonic saline) and stored at −80 °C	Non-pathologic		0.3–9	0.39–2.04		0.15–0.025	Plate-plate	[59]
			CF			0.54–2.25		0.24–0.03		
			COPD			1.4–4.5		0.41–0.04		
		Stored at −40 °C	CF		0.01–10			100–0.05	Cone-plate	[86]
			CF	0.1–10		0.5–1.5	0.5–0.8		Cone-plate	[87]
		Stored at −20 °C	CF		1	9–47	10–58		Cone-plate	[88]
	Airway	Collected by endotracheal tube method and stored at −20 °C	Non-pathologic	0.2–40		3–10		20–0.4	Cone-plate	[89]

**Table 2:** Examples of commonly used immortalized airway epithelial cells and primary cell models.

Cell Type	Cell Name/Species	Phenotype	ALI Culture	TEER (Ω·cm^2^)	TJ Formation	Cilia	Mucus Production	Ref
Immortalized cells	BEAS-2B	Bronchial	No	-	-	-	-	[130]
	NuLu-1	Bronchial	Yes	650–700	Yes	No	No	[132]
	NCI-H292	Bronchial	No	-	No (expresses desmosomes and adherens junctions)	-	No (expresses mucin)	[133]
	NCI-H358	Bronchio-alveolar	No	-	-	-	No (expresses SP-A)	[134]
	16HBE14o-	Bronchial	Yes	150–250	No	No	No	[135,136]
	CFBE41o-	Bronchial mutated homozygote F508del	Yes	150–250	No	No	No	[138]
	A549	Alveolar	No (possible)	150	-	No	-	[140]
	Calu-3	Bronchial	Yes	400–800	Yes	Yes	Yes	[143]
Primary cells	Porcine	Bronchial	Yes	500–1000	Yes	Yes	Yes	[151]
	Sheep	Tracheal	Yes	200–1000	Yes	Yes	Yes	[152]
	Mouse	Tracheal	Yes	1000–2000	Yes	Yes	Possible	[153]
	Bovine	Bronchial	Yes	500–1400	Yes	Yes	Yes	[154]
	Human	Bronchial, tracheal, CFTR mutated or not, iPSC, etc.	Yes	800	Yes	Yes	Yes	[155]
